# Using the Implementation Research Logic Model as a Lens to View Experiences of Implementing HIV Prevention and Care Interventions with Adolescent Sexual Minority Men—A Global Perspective

**DOI:** 10.1007/s10461-022-03776-5

**Published:** 2022-08-10

**Authors:** LaRon E. Nelson, Adedotun Ogunbajo, Gamji Rabiu Abu-Ba’are, Donaldson F. Conserve, Leo Wilton, Jackson Junior Ndenkeh, Paula Braitstein, Dorothy Dow, Renata Arrington-Sanders, Patrick Appiah, Joe Tucker, Soohyun Nam, Robert Garofalo

**Affiliations:** 1 School of Nursing, Yale University, New Haven, CT USA; 2 Center for Interdisciplinary Research on AIDS, School of Public Health, Yale University, New Haven, CT USA; 3 Yale Institute of Global Health, Yale University, New Haven, CT USA; 4 Department of Epidemiology, Harvard T.H. Chan School of Public Health, Boston, MA USA; 5 Department of Prevention and Community Health, Milken Institute School of Public Health, George Washington University, Washington, DC USA; 6 Department of Human Development, State University of New York at Binghamton, Binghamton, NY USA; 7 Center for International Health, Ludwig Maximilian University of Munich, Munich, Germany; 8 Epidemiology Division, Dalla Lana School of Public Health, University of Toronto, Toronto, ON Canada; 9 Department of Pediatrics, School of Medicine, Duke University, Durham, NC USA; 10 Division of Adolescent/Young Adult Medicine, School of Medicine, Johns Hopkins University, Baltimore, MD USA; 11 Youth Alliance for Health & Human Rights, Ashanti Kumasi, Ghana; 12 Division of Infectious Diseases, University of North Carolina Chapel Hill, Chapel Hill, NC USA; 13 Division of Adolescent Medicine, Feinberg School of Medicine, Northwestern University, Chicago, IL USA; 14 Department of Social & Behavioral Sciences, School of Public Health, Yale University, New Haven, CT USA; 15 School of Nursing, University of Rochester, NY Rochester, USA

**Keywords:** HIV, Implementation science, Sexual minority men, Global health, Adolescents

## Abstract

Adolescents and sexual minority men (SMM) are high priority groups in the United Nations’ 2021 − 2016 goals for HIV prevention and viral load suppression. Interventions aimed at optimizing HIV prevention, testing and viral load suppression for adolescents must also attend to the intersectional realities influencing key sub-populations of SMM. Consequently, there is not a robust evidence-base to guide researchers and program partners on optimal approaches to implementing interventions with adolescent SMM. Using a multiple case study design, we integrated the Implementation Research Logic Model with components of the Consolidated Framework for Implementation Research and applied it as a framework for a comparative description of ten HIV related interventions implemented across five countries (Ghana, Kenya, Nigeria, Tanzania and United States). Using self-reported qualitative survey data of project principal investigators, we identified 17 of the most influential implementation determinants as well as a range of 17 strategies that were used in 90 instances to support intervention implementation. We highlight lessons learned in the implementation research process and provide recommendations for researchers considering future HIV implementation science studies with adolescent SMM.

## Introduction

Worldwide, adolescents represent a growing share of people living with HIV. In 2020, 410,000 adolescents between the ages of 10–24 years were newly infected with HIV. AIDS is the leading cause of death among adolescents in sub-Saharan Africa and the second leading cause of death among adolescents across the world. 1 However, a recent review of HIV trends across 25 sub-Saharan African countries showed a promising downward trend in HIV prevalence among both male and female adolescents. 2 The study also found a difference in HIV prevalence based on population density, with adolescents residing in urban areas having at least 1.5 times higher HIV prevalence than counterparts residing in rural areas. 2 All in all, given that Africa is the only continent where the youth population continues to rise and accounts for the majority of new HIV cases, prioritizing adolescents in the HIV response in sub-Saharan Africa is of the utmost importance.

Sexual minority men (SMM) are disproportionately affected by the HIV epidemic 3. While SMM in Africa have relatively high HIV testing rates, they differ vastly by country. HIV testing and status awareness were significantly lower in countries with the most severe anti-LGBT (lesbian, gay, bisexual, and transgender) legislation compared with countries with the least severe legislation 4. Additionally, African SMM living with HIV have relatively low rates of engagement in care (15–40%) and viral load suppression (25%). 4.

Adolescent SMM are confronted with stigma, discrimination, and violence due to their sexual orientation, HIV status, gender expression and other stigmatized identities, which may hinder their ability to access HIV prevention and care services in various African countries. 5 Additionally, studies have found that adolescents have low knowledge of HIV risks behavior and HIV testing rates, and have limited access to comprehensive sex education, all of which may predispose them to HIV infection. 6–8 While pivotal work has been conducted to address HIV in adolescents and SMM in sub-Saharan Africa, there is not a robust evidence-base on HIV-related interventions and programs to engage adolescent SMM in these settings.

Various health interventions have been implemented to curb the spread of HIV among adolescents in sub-Saharan Africa 9, but there is limited research on best practices for HIV programming for adolescent SMM. A systematic review of school-based interventions to reduce sexual risk taking and sexually transmitted infections (STIs) among adolescents in sub-Saharan Africa found that behavioral interventions resulted in a significant decline in unintended pregnancies and an increase in HIV knowledge, condom use, and attitudes towards HIV testing. 10 Another review found that provider initiated and home-based testing and counseling were the most acceptable strategies for increasing HIV testing and counseling behaviors among adolescents in sub-Saharan Africa. 11 A study that documented the process through which youth were engaged in developing an implementation science agenda for HIV testing and care linkage for adolescents in sub-Saharan Africa did not highlight the unique needs and barriers faced by SMM in these settings. 12.

This paper thus seeks to describe the experiences of implementing HIV prevention and care interventions with adolescent SMM in a global perspective. Using the Implementation Research Logic Model as a framework and complemented by the Consolidated Framework for Implementation Research, we describe the determinants encountered during the implementation of HIV prevention and care interventions with adolescent (including sexual minority men), the strategies used to address the determinants, and the types of both implementation and clinical outcomes targeted. We highlight the key lessons learned in the process of conducting this research and provide recommendations for researchers considering future HIV implementation science studies with adolescent SMM in sub-Saharan Africa.

## Methods

### Guiding frameworks: Implementation Research Logic Model and Consolidated Framework for Implementation Research

This analysis is guided by two complementary models that provide an integrated lens through which to examine the selected cases: the Implementation Research Logic Model (IRLM) 13 and the Consolidated Framework for Implementation Research (CFIR) 14. The choice of these two models was based on the need to use tools with specifications to implementation science and that provide rigor and transparency as well as to ensure complementarity in the description of various aspects of the cases 13.

The IRLM is an organizing framework that operationalizes the relationship between its principal components: clinical innovations, implementation determinants, implementation strategies, mechanisms of action and outcomes. 13 In the IRLM, the clinical innovation represents the evidence-based tool for which efficacy has already been firmly established, but for which improved implementation impact is desired. 13 Common clinical innovations in HIV prevention and treatment research include HIV testing (efficacy for determining the presence of HIV), anti-retroviral medication (ARV) for pre-exposure chemoprophylaxis (PrEP) and ARV for HIV treatment. Determinants are factors that can either impede or accelerate the implementation of the clinical innovation. The implementation determinants component of the IRLM incorporates elements of CFIR which provides more detail. 13,14.

The CFIR is a list of constructs that are understood to impact the adoption of evidence into organizational settings. 14 These constructs are organized into five domains. The first is the *characteristics of the clinical innovation* itself and the ways in which it is more or less suitable for implementation. The *outer setting* refers to factors in operation external to the implementing organization that can have influence on the adoption of the clinical innovation, such as local politics, as well as state and municipal policies that govern organizational practices. The *inner setting* refers to the factors within the implementing organization that can influence adoption of the clinical innovation, for example: physical infrastructure, organizational climate and interpersonal workplace dynamics. The *characteristics of individuals’* domain is concerned with the variations in motivation, capacities and performance among the people within the organizations that have substantive involvement in the implementation process. The fifth domain, *process*, are the set(s) of administrative practices that are needed to be able to facilitate and sustain the adoption of the clinical innovation.

There is an array of constructs under each of the five CFIR domains that may be used to understand how specific factors may be influencing implementation. The implementation strategies component of the IRLM, also commonly referred to as “interventions”, are the activities taken to enable the incorporation of the clinical innovation into the organization’s standard practice. 13 The implementation strategies in this paper are presented in connotation with the above domains of the CFIR. IRLM mechanisms of action accommodates information regarding the pathways through which the strategies are hypothesized to have effects on the outcomes specified in the model for a given study. 13 The final IRLM component are the outcomes. Outcomes are the actual output effects of deploying the implementation strategies in support of the adoption of the clinical innovation. 13,15 Outcomes of interest can include the implementation, service and/or clinical effects. 15.

### Design

We conducted a multiple case study of research projects focused on HIV prevention and treatment with adolescents. The cases were drawn from two National Institutes of Health (NIH) sponsored research networks of adolescent HIV implementation science research teams from across the globe. The first network was the *Adolescent HIV Prevention and Treatment Implementation Science Alliance* (AHISA). The AHISA network is sponsored by the NIH Fogarty International Center through its Center for Global Health Studies. AHISA consists of 26 teams led by principal investigators who have active NIH-funded research across 11 countries: Botswana, Ghana, Kenya, Malawi, Nigeria, Rwanda, South Africa, Tanzania, Uganda, Zambia and Zimbabwe. The goal of AHISA is to optimize the use of evidence to remedy obstacles to effective implementation of programs for HIV testing, prevention, and treatment in adolescents. The second network was the *Prevention and Treatment through a Comprehensive Care Continuum for HIV-affected Adolescents in Resource Constrained Settings* (PATC^3^H)—a network sponsored by the Eunice Kennedy Shriver National Institute of Child Health and Human Development, the National Institute on Minority Health and Health Disparities, the NIH Office of Behavioral and Social Sciences Research and the NIH Office of AIDS Research. PATC^3^H consists of eight clinical research teams led by NIH-funded PIs across seven countries: Brazil, Kenya, Mozambique, Nigeria, South Africa, Uganda, and Zambia. The goal of PATC^3^H is to accelerate scientific advances in the development of high-impact public health interventions for adolescents at risk for HIV as well as adolescents living with HIV. We also identified two research projects from outside the AHISA and PATC^3^H networks (including one which is also outside of sub-Saharan Africa), for the purposes of comparing and contrasting these external cases to the in-network cases. Although most of the projects in this multiple case study are members of the AHISA and PATC^3^H research networks, the networks are not the cases. The cases being studied here are the individual research projects.

### Settings and cases

The cases are a convenience sample of studies that were selected because they either focused on SMM, or because they had implications that could potentially inform intervention implementation research with SMM in sub-Saharan Africa. We administered a structured survey to academic researchers in the AHISA and PATC^3^H networks with experience implementing HIV-related intervention with adolescents in sub-Saharan Africa. The survey included: (1) description of the intervention that was implemented, (2) implementation outcomes (target behaviors and clinical outcomes), (3) facilitators of intervention implementation, (4) barriers to intervention implementation, (5) influence of implementation outcomes on HIV service delivery, (6) lessons learned, and (7) recommendations for HIV implementation science approaches and intervention development with adolescent SMM. Our case definition was any HIV intervention research project conducted with adolescents that either was implemented for specific impact on SMM as a population or whose project focus had potential implications (direct or indirect) that could be applied to SMM. All selected projects would have been conducted within the past 8 years. The country settings are diverse, representing the eastern (e.g., Kenya, Tanzania) and western (e.g., Ghana, Nigeria) sub-regions of sub-Saharan Africa as well as the United States.

### Ghana

Ghana is a democratic republic in the western region of Africa, where SMM have 18% HIV prevalence 16. HIV prevention implementation work among SMM in Ghana is centered in its two largest metropolitan areas: Accra and Kumasi. Accra is Ghana’s largest city and also has the highest prevalence of HIV (34%) among SMM. Kumasi is a major trading cross-roads of domestically produced goods due to its geographic location in the center of the country. It has the second highest HIV prevalence among SMM, with an estimate of 17%^16^.

#### Case 1

*Auntie’s Corner*. This is a smartphone-based symptom self-monitoring intervention for adolescent and adult SMM living with HIV. Based on self-determination theory, the web-app enables decentralized access to clinical and psychosocial support using a secure bi-directional messaging system between the men and a team of registered nurses. Auntie’s Corner also allowed men to complete a structured symptom survey that could be evaluated by nurses online. 17 In addition to HIV symptom management, Auntie’s Corner’s goals were to support HIV treatment adherence and viral suppression.

#### Case 2

*HIV Education, Empathy and Empowerment (HIVE*^*3*^). HIVE^3^ is a smartphone-based intervention designed to counteract intersectional stigma by providing three forms of peer support online: informational, emotional, and affirmation. A peer is a person internal to the community, with similar demographics and specific knowledge from lived experience. 18–20. Peer mentors provide emotional and affirmational support through attentive listening and encouragement and informational support through factual input and relevant resources. Support is provided via phone-based text messages and/or via voice contact.

#### Case 3

*Nyansapo.* This is a group-based behavioral intervention, modeled on the Many Men, Many Voices (3MV) intervention, 21 but adapted for use in Ghanaian sociocultural context. 22,23 3MV is listed in the U.S. Centers for Disease Control’s Compendium of Evidence-Based Interventions and is designated as a ‘best-evidence’ intervention for efficacy in reducing the number of sexual partners, episodes of condomless anal sex and increasing HIV testing 21. Nyansapo is delivered in seven sessions and is led by a team of peer co-facilitators who have previously had to experience Nyansapo. 23.

### Nigeria

Nigeria is the most populous country on the African continent, with a population of over 200 million. It is estimated that 1.7 million people in Nigeria are living with HIV. In 2020, Nigeria had approximately 86,000 new cases of HIV infection. One-fifth of all new infections in the country were among 14–24 year-olds. 24 The HIV prevalence in Nigeria is highest among SMM (23%). Overall, Nigeria is close to reaching the 90% threshold of adults living with HIV who are receiving antiretroviral treatment (89%)^24^; however, substantial gaps remain in subpopulations of men (73%) and youth (45%) living with HIV. 24.

#### Case 4

*Innovative Tools to Expand Youth Friendly HIV Testing (iTest).* This youth-centered participatory intervention uses the concepts of open challenges to elicit innovative strategies for HIV self-testing, including a plan for linkage to ongoing prevention services such as PrEP and risk-reduction behavioral counseling. Semi-finalists from the *iTest* open challenge will be selected to receive skills-building apprenticeships to support the enactment of their proposed strategy. The intervention is not the innovative strategy derived from the open challenge, but is the use of the participatory approach of open challenges to generate implementation ideas that resonate with youth.

#### Case 5

*Intensive Combination Approach to Rollback the Epidemic (iCare).* This is an evidence-based mHealth intervention that uses social media outreach strategies to engage Nigerian youth in peer navigation services for entry into the HIV care continuum. The intervention uses a standardized manual of messages that are tailored to the Nigerian sociocultural context and instructions for frequency of posting to popular social media sites. Peer navigators were trained in national HIV testing guidelines, the protection of human rights of individuals engaged in HIV services and maintenance of personal safety. 25.

### Kenya

Kenya is a country in the eastern region of Africa. In 2020, it was estimated 1.4 million Kenyans were living with HIV, 82,000 of whom were under 15 years old. 26 SMM in Kenya have an HIV prevalence of 19%.^27^ In that same year, there were 5,200 new HIV infections among Kenyans under 15 years old. 26 The prevalence of HIV among youth living on the street is also high, with an estimated prevalence of HIV among youth overall at 4.7%—ranging from 2.7% in young men to a high of 8.9% among young women who live on the street. 28.

#### Case 6

*Engaging Street-Involved Youth in HIV Interventions (ESHYI).* This is a multi-level HIV status-neutral combination intervention with six integrated components. The components included peer navigators (current or former street youth), 29 voluntary male medical circumcision (VMMC), 30,31 point-in-time count census enumeration with embedded HIV counseling and testing, 28 comprehensive reproductive health clinical services, HIV education integrated with a matched-savings program, a modified directly observed treatment (mDOT) program for street youth receiving ART for treatment or PrEP, tuberculosis prevention or treatment, and antibiotics incentivized through a daily hot meal.

#### Case 7

*Peer Navigator Project (PNP).* The PNP is a longitudinal, multi-site, study designed to test the adaptation and scale-up of Peer Navigators in three cities in Canada (Toronto, Montreal, and London), and three cities/townships in Kenya (Eldoret, Huruma, Kitale) to increase uptake and utilization of HIV services (prevention, testing, treatment). Phase 1 of this study was dedicated to engaging a range of stakeholders in assessing the appropriateness and acceptability of Peer Navigators (PN) to support this population in HIV services, adaptations needed, and the barriers and facilitators associated with accessing HIV services in these locations.

### Tanzania

Tanzania is also in Africa’s eastern region and it borders Kenya to the north. The estimated HIV prevalence in 4.7% in the general population of adults 32, 1.1% in young men 32 and approximately 8% in SMM. 33 The adult HIV incidence rate is 201 per 100,000. 32 The most recent epidemiological data indicate that there were 20,000 new HIV infections among adult men. 32 Only 45% of men living with HIV are aware of their status, while 86% of men living with HIV are on ART and 84% of those men have suppressed HIV viral load 34—both of which are in reach of the UN’s goals of 90-90-90. 34.

#### Case 8

*The Voice of Youth/Sauti ya Vijana (SYV).* This is a multi-component, multi-level behavioral intervention to support psychological wellness among youth living with HIV by building their skills-capacity to self-manage common mental health challenges. 35 It includes a group component designed for youth and their caregivers and a youth-specific the individual-level component. 35 SYV is culturally-grounded and is tailored to the cognitive developmental stage of adolescents. 35 It is built on three evidence-based clinical approaches to mental health treatment and promotion: Trauma-Informed Cognitive Behavioral Therapy, Interpersonal Psychotherapy and Motivational Interviewing. 35,36.

#### Case 9

*Self-Testing Education & Promotion (STEP).* The STEP intervention is a community-based strategy to enhance the uptake of HIV self-testing among Tanzanian men. 37 It includes a mHealth component that facilitates rapid post-test engagement by a health worker for linkage to either HIV prevention or HIV treatment services. 37 The initial linkage and follow-up with the health provider can occur in the community. 37 The community linkage to a nurse or other community health worker minimizes potential logistical barriers involved when the post-test follow-up options are limited to in-person clinic visits. 17,37 The STEP intervention is a case external to the AHISA and PATC^3^H networks.

### United States

In the United States SMM are disproportionately represented in HIV incidence and prevalence 38. In 2018, the majority (70%) of new HIV diagnoses in the US were among SMM 39. Black SMM are also disproportionately represented in new HIV diagnoses, comprising 37% of the new HIV diagnoses in SMM—a number that is more than triple their population size (12%) among SMM 39. Most (63%) Black SMM living with HIV reside in the southern US 39. Moreover, one-fifth of new HIV diagnoses in the US were among youth, most of whom were SMM 40.

#### Case 10

*Coach-based Mobile Enhanced Intervention (MEI).* This is a case-management intervention that is tailored and enhanced for young Black SMM with unsuppressed HIV viral load. 41 This is an individual-level intervention that provides weekly telephone and app-based coaching support that is anchored by structured quarterly in-person meetings with the coach. 41 The coaching support is geared to enhancing young Black SMMs progression along the HIV care continuum, including linkage, treatment initiation, retention, adherence and viral suppression. 42 The MEI case is external to the AHISA and PATC^3^H networks and is the only case selected outside of the sub-Saharan African region.

### Data Collection

The principal investigators (PIs) for each intervention were contacted by the first and second authors and instructed to complete a brief unstructured six question survey about their experiences implementing HIV interventions with adolescents. The survey was based on the IRLM and asked the PIs to: (1) describe the intervention and its implementation setting, (2) identify the top three barriers and top three facilitators (i.e., determinants) to implementing their interventions, (3) describe any strategies they used to minimize the implementation barriers or to optimize the facilitators and (4) specify the outcome(s) that they attempted to impact through their intervention. The survey then asked the PIs to (5) provide lessons learned from their implementation experiences, with specific attention to how those lessons can be applied to strategies to improve the implementation of HIV prevention and care interventions with adolescent SMM. Finally, each PI was asked to (6) offer recommendations for future research on HIV implementation science with adolescent SMM.

### Analysis

Data from the surveys were subjected to qualitative content analysis 43 using a visual data display Table 44 constructed in Microsoft Word. The determinants (implementation barriers and facilitators) identified in the PI surveys were coded using corresponding CFIR constructs. The survey responses were unstructured qualitative data that were not written in the exact language of CFIR constructs; therefore, the self-reported responses were coded as the CFIR construct to which they most closely matched. The CFIR-coded determinants were then grouped together under the relevant CFIR domains that they reflected 14. The self-reported strategies used to minimize barriers and/or optimize facilitators to implementation were compared against the compilation of implementation strategies from the Expert Recommendations for Implementing Change (ERIC) project 45. The ERIC compilation is a comprehensive, evidence-informed list of 73 common implementation strategies that were derived using a delphi process. The self-reported outcomes were categorized using nomenclature proposed by Proctor and Powell on outcomes, specifically implementation focused outcomes, service outcomes and clinical (patient-level) outcomes 15. The outcomes were summarized in a word table along with information on the intervention and implementation setting.

## Findings

### Descriptive

A descriptive summary of the ten (10) cases is provided in Table I, including each case’s country’s socio-epidemiological context along with the intervention(s) conducted in the country. The cases focused on improving the implementation of a range of clinical innovations (the evidence-based tool for which efficacy has already been firmly established). HIV testing was the most frequent clinical innovation (n = 7), followed by ART (n = 6) and condom use (n = 2). Only one case included implementation of sexually transmitted infection (STI) testing as a clinical innovation. Similarly, only one case included implementation of PrEP as a clinical innovation. None of the cases in Africa focused on PrEP implementation. Although five of the nine cases in sub-Saharan Africa focused on improving the impact of clinical innovations specifically among SMM, five of the cases are in the West African region—and most of those cases (n = 3) are in Ghana. The cases represented a wide variety of interventions used to support implementation of the clinical innovations. There were two pairs of cases that had conceptual overlap in their interventions. Conceptual overlap refers to interventions that utilized similar approaches to improve an HIV-related outcome. These included overlap in their use of online coaching support for adolescent SMM living with HIV (Case 3 - Auntie’s Corner and Case 10 - MEI) and overlap in HIV status-neutral peer navigation (Case 5 - iCare and Case 7 - PNP).

**Table I:** Summary of adolescent HIV implementation science cases and characteristics

Project (PI)	Country (City)	Setting	Population Focus	Clinical Innovation	Implementation Intervention	Outcomes	
						**Implementation**	**Clinical**
Auntie’s Corner (Nelson)	Ghana (Accra /Kumasi)	Mobile (home-based)	SMM living with HIV	HIV ART	Online HIV symptom self-monitoring and nurse coaching	Acceptability Feasibility	HIV viral load suppression
HIVE^3^ (Nelson)	Ghana (Accra /Kumasi)	Mobile (home-based)	SMM	HIV Testing	Online peer support	Acceptability Appropriateness Feasibility	HIV testing frequency
Nyansapo (Nelson)	Ghana (Accra /Kumasi)	Community-based organization	SMM	Condoms HIV Testing STI Testing	Group-based behavioral intervention	Acceptability Feasibility	HIV testing frequency Condom use
iCare (Garofalo)	Nigeria (Lagos)	Mobile (home-based)	SMM	HIV Testing HIV ART	Social media and peer navigation	Adoption Effectiveness Reach	HIV testing frequency Linkage to HIV Care
iTest (Tucker)	Nigeria (Lagos)	Mobile (home-based)	SMM	HIV testing	Training for youth friendly clinical services	Adoption Effectiveness Reach	HIV testing frequency
ESYHI (Braitstein)	Kenya (Eldoret)	Community and facility-based	Street-connected youth	HIV Testing HIV prevention HIV ART	Peer navigators, point-in-time count, VMMC, mDOT and integrated HIV/RH clinical services	Acceptability Adoption Cost Feasibility Sustainability	HIV testing frequency ART or PrEP initiation ART adherence
PNP (Braitstein)	Kenya (Eldoret/ Huruma/ Kitale)	Community and health facility-based	Street-connected youth	HIV Testing HIV prevention HIV ART	Peer navigators	Acceptability Adoption Appropriateness Cost Feasibility	HIV testing frequency ART or PrEP initiation ART adherence
Sauti ya Vijana/ Voice of Youth (Dow)	Tanzania (Moshi)	Hospital based HIV clinic	Youth living with HIV	HIV ART	Mental health promotion and life skills-building	Feasibility Acceptability	Condom use HIV RNA HIV viral load suppression
STEP (Conserve)	Tanzania (Dar es Salaam)	Community-based organization	Heterosexual Men	HIV testing	Community-based HIV self-testing	Feasibility Acceptability	HIV testing frequency
MEI (Arrington)	United States (Baltimore)	Mobile (home-based)	SMM	Condoms HIV PrEP HIV ART	Mobile enhanced coaching and case- management	Fidelity	Condom use PrEP uptake HIV viral load suppression

Feasibility and acceptability were the most frequently studied implementation outcomes—appearing in seven of the cases. Appropriateness was only studied in HIVE^3^ and PNP—both of which are peer-based interventions. Fidelity was only studied in MEI. The two cases in Nigeria (iCare and iTest) both studied reach, effectiveness and adoption as their implementation outcomes of interest. Similarly, only the HIVE^3^ and MEI interventions reported also studying service outcomes (not shown in Table I). HIVE^3^ examined satisfaction of peer support based on the peer support evaluation inventory while MEI examined retention in medical care indicated by > 2 medical appointments in a 12-month period. Consistent with the clinical innovations targeted for implementation, HIV testing (n = 7), HIV viral load suppression (n = 3) and condom use (n = 3) were the most frequent clinical outcomes studied across the cases.

### Implementation determinants

The self-reported implementation determinants and their CFIR domains are shown in Table II. Overall, there were fewer barriers to implementation than facilitators. Across the cases there were only five implementation barriers identified from three of the CFIR domains: outer setting, inner setting and process. In the *Outer Setting* domain, “patient needs and resources” were only barriers to implementation among cases that focused specifically on SMM (Nyansapo, iCare, iTest and MEI). “External policies and incentives” were identified as barriers by all the cases in Ghana, Nigeria, and Kenya but none of the other country cases. In the CFIR *Inner Setting* domain, the “tension for change” determinant was identified as a barrier in at least one case in every country except the United States. The “relative priority” of the intervention among national health funders and international donor agencies was identified as an implementation barrier in both of the Kenyan cases. This implementation barrier-type was reported across two cases in Ghana (Auntie’s Corner and HIVE^3^), both cases in Nigeria (iCare and iTest) and one in Tanzania (SYV). In the process domain, “engaging external change agents ‘’ was the only barrier to implementation reported (ESYHI). Some cases did not report any barriers to implementation. For example, neither of the two cases in Tanzania reported implementation barriers in the outer setting.

**Table II:** Cross-case distribution of implementation determinants and implementation strategies

CFIR Implementation Domain and Implementation Determinants			Implementation Strategies Used									
			**Ghana**			**Nigeria**		**Kenya**		**Tanzania**		**United States**
			**Auntie’s Corner**	**HIVE** ^**3**^	**Nyansapo**	**iTest**	**iCare**	**ESYHI**	**PNP**	**STEP**	**SYV**	**MEI**
**Barriers**	**Outer Setting**											
	1.	Patients’ needs and resources	—	—	AD	MM	MM	—	—	—	—	AP, NW, TS
	2.	External policies and incentives	BC	AD, BC	BC	BC, CM	BC, CM	NW, SC	NW, SC	—	—	—
	**Inner Setting**											
	3.	Tension for change	BC, CM	BC, CM	—	BC, CM	BC, CM	—	—	—	AR	—
	4.	Relative priority	—	—	—	—	—	AR, NW	AR, NW	—	—	—
	**Process**											
	5.	Engaging external change agents	—	—	—	—	—	NW	NW	—	—	—
**Facilitators**	**Intervention Characteristics**											
	1.	Intervention source	IP, OC	OC	YY	—	—	—	—	—	—	—
	2.	Evidence strength and quality	YY	YY	YY	MM	MM	—	—	CM, MM	YY	YY
	3.	Relative advantage	YY	YY	—	—	—	—	—	—	—	—
	4.	Adaptability	AD	—	—	—	EM, TA	—	—	—	—	—
	**Outer Setting**											
	5.	Patients’ needs and resources	YY	IP	IP, AP	AB, EM, IP	AB, EM, IP	IP, TS, SC	IP, TS, SC	CM, IP	AB, IP	IP
	6.	Peer pressure	CM	—	YY	—	—	—	—	—	—	—
	7.	Cosmopolitanism	—	—	—	—	—	NW	NW	—	—	—
	8.	Networks and Communication	—	—	—	—	—	NW	NW	—	—	—
	**Inner Setting**											
	9.	Compatibility	TA	AF, TA	YY	—	EM, TA	—	—	MM	—	—
	10.	Relative priority	YY	YY	—	—	—	—	—	—	—	—
	11.	Leadership engagement	BC	—	BC	BC	BC	IP	IPP	BC	—	—
	**Process**											
	12.	Reflecting and evaluating	AF, OT, TA	AF, OT, TA	AF, TA	—	—	—	—	CD	—	—

There were 12 determinants identified as facilitators from four of the CFIR domains. In the *Intervention Characteristics* domain, “intervention source” and “relative advantage” were only facilitators for cases in Ghana. “Evidence strength and quality” was identified as a facilitative determinant in eight cases. In the *Outer Setting* domain, “patient needs and resources” was identified as a determinant that facilitated implementation in all ten cases; however, “peer pressure” was only an implementation facilitator among cases in Ghana (Nyansapo and Auntie’s Corner). The *Inner Setting* domain included the determinants of “compatibility” and ‘leadership engagement” which were facilitators for cases in Ghana, Nigeria and Tanzania. Kenya was the only country among the cases to identify “cosmopolitanism” and “networks and communication” as implementation facilitators (Case 6 - ESYHI and Case 7 - PNP). The “relative priority” determinant was only identified as facilitator in Ghana. No *Inter Setting* facilitators were reported in the American case. The CFIR domain with the fewest number of identified determinants was the *Process* domain. The determinant of “reflecting and evaluating” was the only construct identified in this domain. This determinant was reported as a facilitator among all the cases in Ghana; however, it was only reported in one the cases outside of Ghana (Case 9 – STEP; Tanzania).

### Implementation strategies

The survey identified a total of 17 strategies that were used to support the implementation of the HIV-related clinical innovations (HIV testing, condoms, PrEP and ART) among the ten cases. Table III presents the ERIC nomenclature for the 17 self-reported implementation strategies derived from the survey. The table includes case-specific examples of how the implementation strategy was operationalized. The 17 strategies in Table III correspond to Table II which displays the application of each strategy to the range of implementation determinants identified across the cases. These 17 strategies were applied 90 different times to address the identified determinants. The most frequently used implementation strategies were “build a coalition” (n = 13), “network weaving” (n = 11), “involve patients and consumers” (n = 8), “conduct an education meeting” (n = 7), “use mass media” (n = 6), “provide technical assistance” (n = 6) and “audit and feedback” (n = 4). Not all implementation determinants that were identified had a corresponding implementation strategy applied to address it. Of the 13 instances where determinants were identified with no corresponding implementation strategy (noted in Table II as “YY”), all 13 were determinants that were reported as facilitators of intervention implementation. Most of these instances (11 of 13) occurred among cases in Ghana. These “YY” instances were also concentrated in the CFIR domain of *Intervention Characteristics*, accounting for 60% of “YY” occurrences. Within each case there were multiple combinations of implementation strategies used. Additionally, some individual implementation determinants within a case had multiple combinations of implementation strategies applied to them.

**Table III:** Inventory of strategies used to minimize barriers and optimize facilitators of intervention implementation

Implementation Strategies (n = 17)		
**ERIC Taxonomy**	**ERIC Definition**	**Implementation Example**
Use advisory boards (AB)	Convene a stakeholder group to provide feedback and guidance on how to optimize implementation efforts.	*Sauti ya Vijana*: Youth advisory group provided input on the low acceptability and feasibility of integrating this topic due to perceived normative stigma towards SMM
Promote adaptability (AD)	Identify how intervention to support the use of a clinical innovation can be locally tailored while maintaining fidelity to the intervention.	*Nyansapo*: Modified intervention to be more inclusive of bisexual behavior since many SMM also have sex with women
Audit and feedback (AF)	Collect performance data over a specified time period and present summaries of the data to providers to aide in their self-evaluation, self-reflection and service improvements.	*HIVE*^*3*^: Patients completed peer support evaluation inventory and the data was used to provide training updates to peer mentors
Prepare patients to be active participants (AP)	Prepare patients to be active engaged in their care, including providing them with tools that will enable them to be most effective at getting their needs met.	*Nyansapo*: Provided anticipatory guidance regarding common hurdles encountered when seeking HIV treatment and used role play to practice navigating the hurdles
Assess for readiness (AR)	Assess organization to determine how its readiness to implement, and factor that may affect implementation	*Sauti ya Vijana*: Deemed the climate too polarized to integrate a topic on SMM
Build a coalition (BC)	Develop supportive relationships with implementation stakeholders	*Auntie’s Corner*: Linked with nurses, local influencers and peers to cooperate together
Conduct local consensus discussions (CD)	Convene local stakeholders for discussions that address the relative importance of the identified problem the relevance of the clinical innovation to the organization.	*STEP*: Conducted discussions to understand the difference in priorities between academic and implementing partners
Conduct education meeting (CM)	Hold meetings targeted toward different stakeholder groups to teach them about the clinical innovation	*STEP*: Conducted meetings to educate stakeholders on the benefits of HIV self-tests
Develop educational materials (EM)	Develop and standardized operating manuals and other supporting materials that facilitate the implementers’ learning of how to deliver the intervention.	*iCare*: Developed intervention manual and frequently asked questions document to support handling unanticipated situations
Involve patients and consumers (IP)	Engage or include patients and families in the implementation effort	*iTest*: Youth led the implementation and were employed as research assistants
Use mass media (MM)	Leverage media platforms to raise awareness of the clinical innovation among a large number of people	*iTest*: Used social media to bring attention the intervention
Promote network weaving (NW)	Identify and leverage durable and functional relationships that are internal and external to the organization to promote implementation of the clinical innovation	*PNP*: Conducted pre-implementation stakeholder engagement and leveraged pre-existing networks and relationships
Provide ongoing consultation (OC)	Provide ongoing expert consultation on the strategies used to support implementation of the innovation	*Auntie’s Corner*: Nurse practitioner provided consults to registered nurse (RN) coaches
Conduct ongoing training (OT)	Conduct training on the intervention at regular intervals and on-demand, as needed	HIVE^3^: Held bi-weekly review sessions to reinforce tenets of the peer support model
Stage scale-up of implementation (SS)	Stage implementation by piloting the strategy on a small before executing a full implementation across a system	*ESYHI*: Piloted intervention prior to implementation at scale
Provide technical assistance (TA)	Develop and use a system to deliver technical assistance focused on implementation using local personnel	*iCare*: Provided TA on navigating functional differences of various social media platforms
Tailor strategies (TS)	Tailor the implementation strategies to minimize barriers and leverage facilitators identified via formative research.	*MEI*: Integrated youth-oriented activities to coaching, such as marketing drop-in sessions as pizza parties instead

## Discussion

In this paper we used the IRLM to examine the experiences of implementing HIV-related interventions among adolescents, with emphasis on adolescent SMM. Ten research projects were used as cases and compared to determine patterns of similarities and dissimilarities between them. In some instances, we compared and contrasted clusters of cases by their country of implementation (e.g., Ghana vs. Nigeria vs. Tanzania). We identified 17 implementation determinants (5 barriers and 12 facilitators) and 17 implementation strategies. The findings from this multiple case study provide important insights for HIV implementation science by providing experiential insights that can be used to inform course corrections to implementation studies that are currently in the field as well as inform the design of implementation studies under development or those that will be conceptualized in the future.

The IRLM has been proposed as a useful tool in the design of research studies 46, including HIV implementation studies 46. We have shown here that it is also a useful tool for distilling information to help understand the implementation research logic of prior studies; thus, allowing for the information to characterized and disseminated for knowledge exchange. Across the cases in this study, we found a stark imbalance between barriers and facilitators of implementing HIV interventions with adolescents. This finding is favorable for implementation and suggests that there may be relatively more advantageous conditions than there are challenges. Moreover, the barriers to implementation were confined to three CFIR domains. Nonetheless, the distribution of the Outer Setting barriers was primarily among cases whose implementation attempted to have direct impacts on adolescent SMM. This was true in the case of the African and American projects. Further, the “external policies and incentives” seemed to have particular salience among these cases. First, we focus on this because it was the most frequently identified barrier (identified in 7 of 10 cases). Second, it was the only barrier that was identified across SSA (east and west) regions. Last, it was also the only barrier that was identified among all the cases that focused specifically on SMM. This highlights the need to consider the intersectional realities of adolescents who are SMM and the way in which policies and practices that marginalize SMM at the individual-level may also be a source of significant complexity that can impede implementation progress. 47,48 Previous studies have documented how HIV stigma intersects with sexual stigma to produce oppressive healthcare organization climates that makes it difficult for SMM to receive safe and high quality medical and nursing services 48–50. Additionally, adolescence is a developmental life stage that may also be stigmatized in ways that can form a tripartite of intersectional HIV, sexual and adolescence stigmas 51,52. This age-related stigma can manifest as dismissal of one’s sexuality as immaturity, confusion, experimentation or rebelliousness 53. These norms of stigma are social processes that can exist latently in an organization’s culture and practices; however, interventions that aim to increase HIV prevention or treatment uptake among adolescent SMM can trigger the activation of these stigmas within implementing agencies and impede outcomes.

There was consistency across country clusters (Ghana vs. Nigeria) in the strategies used to address the implementation barrier “external policies and incentives” in the *CFIR Outer Setting* domain. The implementation strategy of building a coalition was used in all five cases from these two countries. Coalition building is a well-established diplomatic strategy that brings together a diverse cross-section of key stakeholders to identify shared interests 54–57. It has been an effective tool in addressing political barriers to implementing change in organizations and municipal settings 45,58,59. The “building a coalition”strategy may have been especially salient in Nigeria and Ghana because all five cases in the sample were implementing interventions focused on sexual minority men. The social environment in these two countries has not been favorable to sexual minority identities and behaviors ^4,60−62^. In both settings there is either current or pending legislation that *de facto* criminalizes human sexual diversity and proposes severe penalties for individuals or organizations suspected to be in violation of these statutes 63. The political dynamics and actions create an environment of structural stigma that can be translated into policy decisions and institutionalized in organization practices—ultimately undermining the epidemic impact of clinical innovations^42,64−67^. Against this background of political and economic risk for implementing organizations, careful coalition building is a crucial strategy for ensuring progress towards achieving HIV prevention and treatment goals for key populations goals without traversing legal boundaries. There are various models of goal-oriented coalition building for reducing structural-level stigma and organizational-level barriers to implementing clinical innovations 68–74. For example, the Communities That Heal (CTH) intervention used a community-engaged coalition building to help align evidence-based practices for opioid overdose prevention that were aligned with local community norms, practices, resources and statutes across four US states. 71 In the Stigma-Based Solidarity (SBS) model, coalition-building is accomplished through the pursuit of intergroup relations between individuals who perceive themselves as different but have commonalties in their experiences of social marginalization. 70 In an SBS approach, SMM and nurses (most of whom are women) may perceive themselves as different with non-intersecting interests; however, an intergroup identification may be possible based on raising a shared awareness of how femininity is systematically devalued in patriarchal systems and that the same system that devalues their labor and value as nurses also devalues SMM because of the group’s perceived association with “womanliness”. The community-engaged coalition building strategy is not a cross-sectional implementation activity, but a long-term relational strategy that requires ongoing attention and nurturance to maintain its effectiveness.

The findings demonstrate the robust functionality of ERIC implementation strategies 45 which can be used multiple times within an implementation project and can also be used in combination (i.e., multiple strategies) to address a specific implementation determinant. Most of the self-reported CFIR implementation determinants were facilitators. Nonetheless, there was not a discernable pattern of ERIC implementation strategies matched with CFIR determinants. These findings mirror the results from earlier research in which investigators were unable to discern a pattern between implementation determinants and the specific ERIC implementation strategies chosen to address them 75. In one study, 169 implementation science scholars and practitioners were asked to identify a matching strategy (or strategies) that would address specific implementation determinants. 75 Participants selected an average of six implementation strategies per determinant. 75 The current state of the science in HIV implementation research has not yet established benchmarks on any specific number of strategies that can reasonably be expected per implementation determinant. The consistency between our findings and those of the previous study indicate that the field could benefit from best practice guidance on maintaining parsimony in the selection of strategies that are most appropriately matched with a determinant—which will be important for implementation efforts in resource constrained settings. In other sectors, guidance has been developed to aid organizations in identifying and selecting evidence-based interventions 76. This type of guide can be the basis for modeling a similar set of practices that support informed selection of implementation strategies to match the determinants. Even though there will always be some situational factors that warrant uncommon matches between determinants and strategies; guidance on the matching process may contribute to a more cohesive evidence-base in matching strategies and determinants, as well as increase efficiency and effectiveness of HIV implementation science research with adolescents.

Even though there was a not a pattern recognized between determinants and strategies, the cases in the study tended to select strategies that were easy to employ and had high importance. In a concept mapping study to characterize the relationship between ERIC implementation strategies, feasibility and importance, researchers found that the 79 ERIC strategies were distributed across four quadrants on the concept map: I = feasible and important, II = feasible but not important, III = important but not feasible and IV = neither feasible nor important 58. Of the 17 implementation strategies used in this current study, 13 mapped onto quadrant I. The remaining four strategies were either important but not feasible (i.e., use mass media, promote network weaving) or were neither important nor feasible (i.e., provide technical assistance, prepare patients/consumers to be active participants). When viewed against the four quadrants of the concept mapping our findings provide evidence that the most frequently used strategies in this multiple case study were the ones deemed easiest to deploy and most valuable for making progress towards project outcomes. Our findings also revealed that there were some facilitative determinants identified with no corresponding implementation strategy applied to optimize them. While the IRLM helped to elucidate the many facilitative determinants, our study findings highlight the potential for important missed opportunities to leverage facilitative conditions that could be assets in the implementation process. The current available literature is focused on strategies that minimize barriers to implementation^58,75,77−79^ thus leaving a knowledge gap regarding strategies that optimize the effects of facilitators on the implementation process.

### Lessons learned

There were key lessons learned across the nine implementation projects profiles in this multiple case study. First, we learned the importance for implementation projects with youth to include connections between relevant services for youth that increased the perception of coordination quality and the integrity of the continuity in their care. Efforts to reduce service and programmatic fragmentation will contribute to higher engagement and retention of youth—which is an important factor that can influence the interest of implementing partners. Next, our experience across the nine projects in five countries indicate that adolescents living with HIV prefer not to be separated from other adolescents. This is also true among adolescent SMM who understand that there may be a great advantage to learning from the other experiences of adolescents with similar sexual and gender identities, even if they do not share the same health diagnosis. Nearly all of the cases utilized peers in their interventions and identified this as a strength. Peer models should incorporate a youth-centered approach and consider as many aspects of the adolescents’ lives as possible—an approach which will create affinity between the peer and the patients which can be leveraged as source for relational motivation by facilitating authenticity and self-determination 80–82 among adolescents who may otherwise be compelled to conceal aspects of their personhood due to structural-level and organizational-level stigmas. Moreover, despite the challenges that exist for adolescent SMM community they are very determined to make their voices heard and engage in the activities to improve the services for SMM. HIV implementation research should consider elements that build their capacity for collective efficacy in advocating for human rights and access to justice. Finally, we learned that deficit-based approaches to implementation science research contributes to intersectional stigma. Modern approaches will balance behavioral risk-reduction strategies with strength-based strategies that advance emancipation and empowerment. Adolescents are resilient and have the experience and insights to contribute to processes designed to improve clinical outcomes targeted in implementation research.

We offer three recommendations for investigators and practitioners considering future HIV implementation science research The first recommendation is to engage public health agencies (e.g., local, state, district, regional or national), implementing partners and relevant community stakeholders who have worked with adolescents, including adolescent SMM, early and often. These stakeholders will have the keenest insight on what is feasible and acceptable based on their own knowledge of the local sociopolitical environment and on their previous experience in HIV work with adolescent SMM and other youth. The second recommendation is to involve SMM peer educators who are trained in outreach efforts to reach SMM with HIV-related services. While there are some advantages to recruiting non-peer educators, this has the potential to create distance between the non-peer and the adolescents which risks undermining their overall enthusiasm and engagement. The third recommendation is to invest in longitudinal community engagement. We recommend that researchers spend enough time in the country to learn the culture and become familiar with the new stakeholders. This approach is superior to one-time cross-sectional field visits because the government and community stakeholder rosters frequently change based on decisions of new ruling political parties, new management leadership in organizations and/or changes when individuals pursue career opportunities in other organizations or locations. Failure to identify, re-validate, and nurture ongoing relational connections can result in the erosion of important social capital that is necessary for positively influencing an organization’s receptivity to implementing interventions.

There are important limitations to the study presented in this paper. First, we studied cases that were sampled primarily from NIH-sponsored networks of implementation research projects that were identified through a competitive peer-reviewed process. These projects include experts in HIV implementation science that have demonstrated successful partnerships in country. This sample is not representative of the universe of HIV implementation research projects focused on adolescents and likely underrepresents investigators at early stage of developing research-community partnerships, local organizational infrastructure capacity, and local relationships required for successful launch of implementation research. While we found there were relatively few implementation barriers compared to facilitators, we did not quantify the magnitude of the barriers in comparison to facilitators. This could be attributed to recall bias, where study participants might be more likely to recall facilitators rather than the barriers that their intervention program aimed to alleviate. The use of count data may not convey the scale of complexity of the relatively few barriers that we encountered and thus should be interpreted with caution. Only one case in this study had a focus on PrEP. We did not include a case from Africa that aimed to improve the adoption of PrEP, although it is likely that there are some PrEP implementation studies underway (or completed) in Africa. Lastly, we endeavored to represent a diversity of implementation settings by including projects from multiple global regions; however, we recognize that the social and political realties can vary widely within regions and between regions. These limitations notwithstanding, the findings from the study provide important insights to implementation scientists conducting HIV research with adolescent SMM.

## Conclusions

As the scientific community mobilizes to expedite progress towards achieving the 90-90-90 goals outlined by the Joint United Nations Programme on HIV/AIDS (UNAIDS) the role of implementation science has become ever clearer. Additionally, advances in achieving the 90-90-90 goals cannot be achieved without focusing attention on achieving equitable prevention impact in key populations, including adolescents, SMM and adolescent SMM. The IRLM is an important tool that can be used for examining completed implementation research projects to generate cross-case evidence that can inform the design of future impact-focused research. The IRLM and the relevant tools that intersect with it (e.g., CFIR implementation determinants, ERIC implementation strategies) are robust and comprehensive; however, fewer barriers were identified compared to facilitators and the strategies identified to optimize the facilitators were low-complexity but high importance. Dissecting these implementation projects using the IRLM helps to demystify adolescent HIV implementation science and expands its accessibility to researchers and implementers,

*Notes.* AB = advisory board; AD = promote adaptability; AF = audit and feedback; AP = prepare patients to be active participants; AR = assess for readiness; BC = build coalition; CD = consensus discussion; CM = conduct educational meeting; EM = develop educational materials; IP = involve patients and consumers; MM = use mass media; NW = network weaving; OC = ongoing consultation; OT = ongoing training; SS = stage scale up of intervention; TA = technical assistance; TS = tailor strategies; YY = indicates that the implementation determinant applied to this intervention, but no specific strategy was used to influence the determinant.

## Data Availability

Source documentation of principal investigator surveys and qualitative coding are available upon request to corresponding author.
