# Correction to: Using the Implementation Research Logic Model as a Lens to View Experiences of Implementing HIV Prevention and Care Interventions with Adolescent Sexual Minority Men—A Global Perspective

**DOI:** 10.1007/s10461-022-03823-1

**Published:** 2022-09-13

**Authors:** LaRon E. Nelson, Adedotun Ogunbajo, Gamji Rabiu Abu-Ba’are, Donaldson F. Conserve, Leo Wilton, Jackson Junior Ndenkeh, Paula Braitstein, Dorothy Dow, Renata Arrington-Sanders, Patrick Appiah, Joe Tucker, Soohyun Nam, Robert Garofalo

**Affiliations:** 1 School of Nursing, Yale University, New Haven, CT USA; 2 Center for Interdisciplinary Research On AIDS, School of Public Health, Yale University, New Haven, CT USA; 3 Yale Institute of Global Health, Yale University, New Haven, CT USA; 4 Department of Epidemiology, Harvard T.H. Chan School of Public Health, Boston, MA USA; 5 Department of Prevention and Community Health, Milken Institute School of Public Health, George Washington University, Washington, DC USA; 6 Department of Human Development, State University of New York at Binghamton, Binghamton, NY USA; 7 Center for International Health, Ludwig Maximilian University of Munich, Munich, Germany; 8 Epidemiology Division, Dalla Lana School of Public Health, University of Toronto, Toronto, ON Canada; 9 Department of Pediatrics, School of Medicine, Duke University, Durham, NC USA; 10 Division of Adolescent/Young Adult Medicine, School of Medicine, Johns Hopkins University, Baltimore, MD USA; 11 Youth Alliance for Health & Human Rights, AshantiKumasi, Ghana; 12 Division of Infectious Diseases, University of North Carolina Chapel Hill, Chapel Hill, NC USA; 13 Division of Adolescent Medicine, Feinberg School of Medicine, Northwestern University, Chicago, IL USA; 14 Department of Social & Behavioral Sciences, School of Public Health, Yale University, New Haven, CT USA; 15 School of Nursing, University of Rochester, Rochester, NY USA

## Correction to: AIDS and Behavior 10.1007/s10461-022-03776-5

The original version of this article unfortunately contained an error, and it has been corrected with this erratum.

The co-author name of “Robert Garofalo” is misspelled as ““Robert Garafalo” and there appears to be a formatting irregularity on page 4 under “case 2” these corrections are updated.

The original article has been corrected.

